# Metabolic molecular markers of the tidal clock in the marine crustacean *Eurydice pulchra*

**DOI:** 10.1016/j.cub.2015.02.052

**Published:** 2015-04-20

**Authors:** John Stuart O’Neill, Kate D. Lee, Lin Zhang, Kevin Feeney, Simon George Webster, Matthew James Blades, Charalambos Panayiotis Kyriacou, Michael Harvey Hastings, David Charles Wilcockson

**Affiliations:** 1 MRC Laboratory of Molecular Biology, Cambridge CB2 0QH, UK; 2 Bioinformatics and Biostatistics Analysis Support Hub & Department of Genetics, University of Leicester, Leicester LE1 7RH, UK; 3 School of Biological Sciences, Bangor University, Bangor LL57 2UW, UK; 4 Institute of Biological, Environmental, and Rural Sciences, Aberystwyth University, Aberystwyth SY23 3DA, UK

## Abstract

In contrast to the well mapped molecular orchestration of circadian timekeeping in terrestrial organisms, the mechanisms that direct tidal and lunar rhythms in marine species are entirely unknown. Using a combination of biochemical and molecular approaches we have identified a series of metabolic markers of the tidal clock of the intertidal isopod *Eurydice pulchra*. Specifically, we show that the overoxidation of peroxiredoxin (PRX), a conserved marker of circadian timekeeping in terrestrial eukaryotes [1], follows a circatidal (approximately 12.4 hours) pattern in *E. pulchra*, in register with the tidal pattern of swimming. In parallel, we show that mitochondrially encoded genes are expressed with a circatidal rhythm. Together, these findings demonstrate that PRX overoxidation rhythms are not intrinsically circadian; rather they appear to resonate with the dominant metabolic cycle of an organism, regardless of its frequency. Moreover, they provide the first molecular leads for dissecting the tidal clockwork.

## Teaser

O’Neil *et al.* show that overoxidation of peroxiredoxin and the expression of mitochondrial genes follow a tidal rhythm in a marine crustean. These findings could help identify the molecular underpinnings of tidal/lunar clocks.

## Main Text

**Figure 1:**
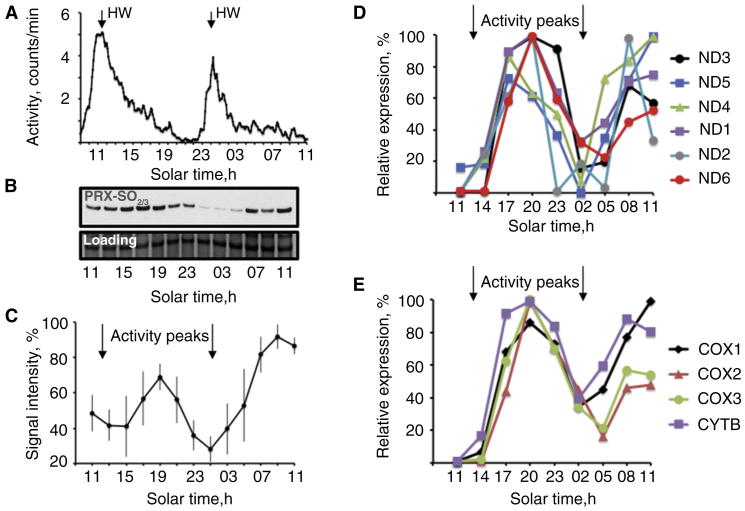
Molecular readouts of the circatidal clock. (A) Section of representative actogram depicting mean spontaneous swimming activity of *E. pulchra* (n = 21, period = 12.5 ± 0.1 h; see [2] for methods) HW: predicted high water on home beach. (B) Representative western blot of overoxidised peroxiredoxin and loading control. (C) Relative intensity of overoxidised peroxiredoxin signal from *E. pulchra* heads collected May 2014 (Group data, mean ± SEM, n = 4 collections, 10 heads per sample) (ANOVA time effect p < 0.005; cosinor period = 14.5 h). See [1] for methods. Arrows indicate peak swimming activity in parallel sample. (D,E) Relative abundance of mitochondrially encoded mRNAs from *E. pulchra* heads (10 per sample), collected over two tidal cycles under free-running laboratory conditions (April 2013). ND, subunits of NADH dehydrogenase; COX, cytochrome oxidase subunits; CYT B, cytochrome B. All profiles were significantly (p < 0.05) rhythmic by cosinor analysis (mean period ± SEM: mRNAs in D = 15.2 ± 0.6 h, E = 15.7 ± 0.8 h). Three mRNAs encoding large and small ribosomal RNA subunits, and ATP synthase subunit had comparable profiles but failed to reach statistical significance. RNA was extracted using Trizol (Life Technologies) and DNAse treated with Turbo DNAfree (Ambion). Libraries for RNAseq were prepared with Illumina TruSeq sample preparation kit, transcriptome was assembled with Trinity (release 2014-07-17), read counts calculated with RSEM 1.9, data annotated in Trinotate (release 2014-07-08), assessed by Transrate 0.3.1 and CEGMA 2.5 and validated with BLAST+2.2.28 against the mitochondrial genome.

To adapt to its intertidal niche, *E. pulchra* expresses a combination of circadian and circatidal behavioural and physiological rhythms, governed by independent and dissociable oscillators with periods of ca. 24 hours and ca. 12.4 hours, respectively [2]. Whereas the circadian clock mechanism parallels those of *Drosophila* and mouse, the mechanisms underlying tidal timekeeping in *E. pulchra*, and other tidally active organisms, are unknown. In overtly circadian organisms, including mice and *Drosophila*, mitochondrial metabolism exhibits daily regulation [3], and PRX proteins exhibit circadian cycles of overoxidation [1,4]. PRX overoxidation cycles may thus represent a conserved, ancestral circadian mechanism [1]. Given that *E. pulchra* has both circadian and tidal clocks, we examined the temporal pattern of PRX overoxidation in heads, which are known to express circadian cycles of gene expression [2]. If PRX overoxidation were an exclusively circadian marker, then a ca. 24 hour cycle would be expected. However, a tidal pattern would not only question current interpretations of PRX overoxidation cycles, but would also reveal a novel molecular substrate for the tidal clockwork. Independent cohorts of *E. pulchra* were collected in 2012 and 2014 (three and four replicates, respectively) from their home beach (Red Wharf Bay, Anglesey, U.K.) and transferred to an activity-recording apparatus to confirm their tidal swimming/rest cycles under free-running conditions [2] (Figures 1A and S1A in Supplemental Information, published with this article online). Heads were then harvested over two further tidal cycles and the extracts subjected to western blotting for PRX overoxidation. In common with samples from a broad range of species [1], we identified a clear band at ∼20 kilodaltons corresponding to the overoxidised PRX monomer that was enhanced by peroxide oxidation (Figures 1B and S1B,D). The intensity of the band in individual blots, and as grouped means of replicates from both 2014 and 2012, exhibited a clear circatidal variation (Figures 1C and S1C). Moreover, the peak was coincident with the phase of reduced swimming activity. A similar inverse relationship with locomotor activity was reported in *Drosophila* [1]. The sustained tidal increase in metabolic rate around high water [5] is followed, therefore, by increased overoxidized PRX. Thus, PRX overoxidation cycles are not exclusively circadian, and are a marker of tidal timing in *E. pulchra*.

PRX overoxidation rhythms are thought to reflect underlying changes in cellular redox/metabolism and proteasomal activity [4,6]. Since overoxidation is tidally regulated in *E. pulchra*, and respiratory rate is also tidal [5], we sought to identify likely molecular/genetic substrates linking these metabolic rhythms. As a first step, we took advantage of the recently published partial mitochondrial genome of *E. pulchra* [7] to interrogate RNAseq data and thereby identify tidally regulated, mitochondrially encoded transcripts. Animals were collected and their circatidal behaviour monitored for two cycles [2]. Heads were harvested every three hours, over two further tidal cycles; RNA was extracted and prepared for RNAseq (conducted at Genomic Core Facility, CRUK, Cambridge).

11 of the 13 known mitochondrially encoded, protein-coding genes were detected, and 10 revealed a clear, statistically significant circatidal pattern of expression (Figure 1D,E). Consistent with their co-regulation, the RNAs peaked with a common phase, coincident with the resting phase of the swimming rhythm, and with PRX overoxidation. Thus, expression of the components of complex I (NADH dehydrogenase) and complex IV (cytochrome c oxidase) are circatidally regulated within mitochondria. Nascent mitochondrial RNA is translated co-transcriptionally. It is likely, therefore, that the RNA rhythms are translated into tidal rhythms of abundance of proteins serving electron transport and oxidative phosphorylation, anticipating the demands of the tidal rest/activity cycle [5].

Our mitochondrial transcriptomic data complement our observation that PRX overoxidation is subject to tidal regulation in a tidal organism. Moreover, these tidal cycles may be functionally related because respiration generates reactive oxygen species that must be detoxified by antioxidants such as PRX in order to protect against oxidative damage. We anticipate that defining the mechanisms underpinning the cycle of mitochondrial transcription will provide an invaluable guide to elucidating the tidal clock. Although we cannot exclude organelle-autonomous timekeeping, by analogy with circadian mechanisms in chloroplasts [8] we consider it more likely that the nuclear genome is pivotal in tidal timing. The transcription of mitochondrial genes is polycistronic, involving mitochondrial RNA polymerase and transcription factors A, B1 and B2. An important question, therefore, is whether the activity of these nuclear-encoded proteins is also tidally regulated. This might occur through rhythmic translocation into the mitochondrion and/or rhythmic nuclear expression. In the latter case, putative *cis*-acting regulatory elements in these nuclear genes (‘tidally responsive DNA enhancers’, TyDEs), analogous to the E-boxes of the circadian clock [9], may be the pivot of a tidal transcriptional feedback loop.

In conclusion, the current observations extend the temporal range over which PRX overoxidation rhythms occur — from 24 hours to 12.4 hours in terms of natural clocks, as well as ultradian as seen in the yeast respiratory oscillation [10]. Thus, PRX oscillations cannot be viewed as an exclusively circadian marker; rather they adopt the frequency of whichever species-typical metabolic program predominates. In addition, by identifying a series of metabolic molecular markers of tidal timing, we have presented an opportunity to unravel, for the first time, the molecular genetic basis of a tidal biological clock.

## Supplemental Information

Document S1. One Figure
