# Supplementary material for: Metabolic molecular markers of the tidal clock in the marine crustacean Eurydice pulchra
Source: Curr Biol. 2015 Apr 20;25(8):R326–7. doi: 10.1016/j.cub.2015.02.052 (PMC4406942; doi:10.1016/j.cub.2015.02.052)
Supplement: Document S1. One Figure [file mmc1.pdf]

Supplemental Information: Metabolic molecular markers of the tidal clock in the marine crustacean *Eurydice pulchra*

Figure S1.

Circatidal rhythms of swimming activity and PRX overoxidation in *Eurydice pulchra*.

A. Section of representative actogram depicting mean spontaneous swimming activity of *E. pulchra* collected October 2012 (n= 15, period= 12.21+0.04h) HW: predicted high water on home beach. B. Representative western blot of overoxidised peroxiredoxin and loading control. C. Relative intensity of overoxidised peroxiredoxin signal from *E. pulchra* heads (Group data, mean+SEM, n=3 collections, 10 heads per sample) (ANOVA time effect $p<0.002$; cosinor period =12.8+0.9h). Arrows indicate peak swimming activity in parallel sample. D. Peroxiredoxin overoxidation signal in control *E. pulchra* heads enhanced by pre-treatment with hydrogen peroxide

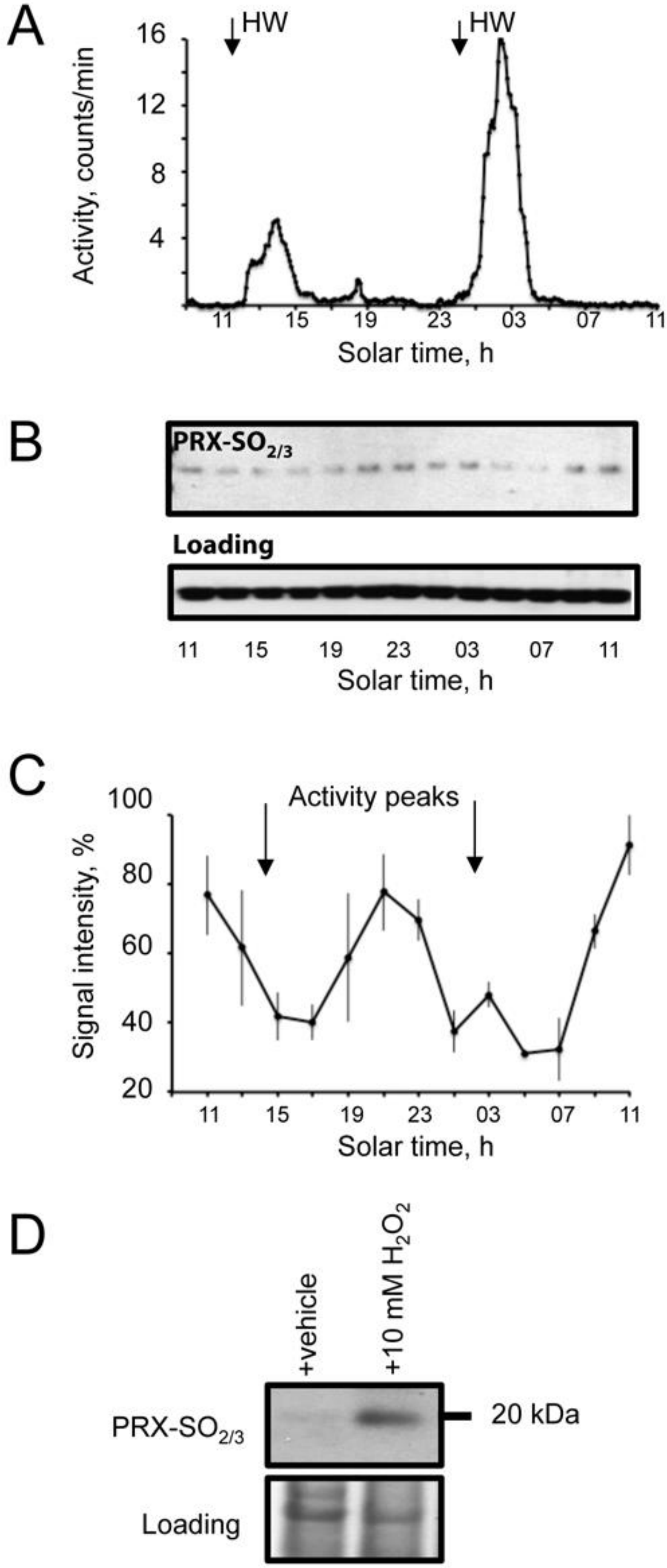

A
HW
HW
Activity, counts/min
16
12
8
4
11
15
19
23
03
07
11
Solar time, h
B
PRX-SO2/3
Loading
11
15
19
23
03
07
11
Solar time, h
C
Activity peaks
Signal intensity, %
100
80
60
40
20
11
15
19
23
03
07
11
Solar time, h
D
+vehicle
+10 mM H2O2
PRX-SO2/3
20 kDa
Loading
